# Effects of micro/nano-ozone bubble nutrient solutions on growth promotion and rhizosphere microbial community diversity in soilless cultivated lettuces

**DOI:** 10.3389/fpls.2024.1393905

**Published:** 2024-04-11

**Authors:** Qinsong Zhao, Jingjing Dong, Shibiao Li, Wenxin Lei, Ake Liu

**Affiliations:** Department of Life Sciences, Changzhi University, Changzhi, China

**Keywords:** lettuce, micro/nano-oxygen nutrient solution, micro/nano-ozone bubble nutrient solution, physiological index, microbial community diversity

## Abstract

Due to its high efficacy as a wide-spectrum disinfectant and its potential for the degradation of pollutants and pesticides, ozone has broad application prospects in agricultural production. In this study, micro/nano bubble technology was applied to achieve a saturation state of bubble nutrient solution, including micro-nano oxygen (O_2_ group) and micro-nano ozone (O_3_ group) bubble nutrient solutions. The effects of these solutions on lettuce physiological indices as well as changes in the microbial community within the rhizosphere substrate were studied. The application of micro/nano (O_2_ and O_3_) bubble nutrient solutions to substrate-cultured lettuce plants increased the amount of dissolved oxygen in the nutrient solution, increased the lettuce yield, and elevated the net photosynthetic rate, conductance of H_2_O and intercellular carbon dioxide concentration of lettuce plants. Diversity analysis of the rhizosphere microbial community revealed that both the abundance and diversity of bacterial and fungal communities in the substrate increased after plant cultivation and decreased following treatment with micro/nanobubble nutrient solutions. RDA results showed that the microbial community in the S group was positively associated with EC, that in the CK and O_2_ groups exhibited a positive correlation with SC, and that in the O_3_ group displayed a positive correlation with CAT and POD. Overall, the implementation of micro/nanobubble generation technology in soilless substrates can effectively increase the lettuce growth and yield, and O_3_ had a more pronounced effect on lettuce yield and quality and the microbial community structure in the substrate than O_2_. Our study would provide a reference and theoretical basis for developing sustainable and green technology for promoting lettuce production and can be a promising alternative to conventional methods for improving crop yields.

## 1 Introduction

Currently, soil disinfection has become widely used in agriculture, which can reduce the occurrence of pests and diseases at the source and improve vegetable quality and yield (Gullino et al., 2022). The use of conventional chemical pesticides and disinfectants generally leads to detrimental effects (Rani et al., 2021). Improper utilization of pesticides can also adversely affect the quality of water, soil and air (Tudi et al., 2021). Consequently, uncomplicated and effective disinfection techniques used for decontaminating less contaminated soil is gaining traction. Recently, ozone has been considered an environmentally friendly method extensively applied in various fields, including shelf life extension of products, deodorization, food, water and air purification, industrial wastewater treatment (Li et al., 2009), preservation of vegetables and fruits (Miller et al., 2013), and rhizosphere soil sterilization (Díaz-López et al., 2022).

Ozone is an allotropic form of oxygen with a strong oxidation potential, and it can decompose into oxygen without harmful residues (Tomiyasu et al., 1985). Compared with alternative methods of soil disinfection, ozone sterilization exhibits rapid disinfection speed and robust sterilization efficacy without inducing pathogenic microorganism resistance (Botondi et al., 2021; Díaz-López et al., 2022). Ozone can target proteins, unsaturated lipids, respiratory enzymes and peptidoglycans in cell membranes, enzymes and nucleic acids in the cytoplasm, and proteins and peptidoglycans in sporangia and viral capsids (Khadre et al., 2006). Due to its rapid decomposition and antibacterial properties, ozone treatment can improve crop quality and antioxidant capacity (Piechowiak et al., 2022) and greatly increase the stress resistance, photosynthetic capacity and yield of tomatoes (Xu et al., 2021). It can also inactivate *Escherichia coli* and mycelia in dry figs, thereby reduce aflatoxin levels (Zorlugenç et al., 2008).

Soil cultivation is the predominant method for crop production and requires regular soil aeration and irrigation; however, numerous pathogenic microorganisms also proliferate in the soil, competing for nutrients with crops and consequently impeding crop growth and defense mechanisms (Das et al., 2022). Soilless cultivation has gradually become as a pivotal component of facility horticulture and the primary mode of factory production for crops owing to its inherent advantages, such as fertilizer conservation, water preservation, enhanced yield potential, superior quality output, and impeccable hygiene standards (Gruda, 2022). Soilless substrate can replace natural soil to anchor crop roots, and supply essential nutrients through drip or trickle irrigation to ensure optimal growth and development (Asaduzzaman et al., 2015; Fields et al., 2021). Moreover, soilless substrate cultivation not only prevents soil disturbance and reduces land pressure and degradation, but also promotes crop intensification through year-round production (Gebreegziher, 2023). Compared to traditional soil cultivation, the composition and concentration of nutrient solution and growth media can be precisely controlled in soilless cultivation, and the yield and quality of horticultural crops can also be greatly improved (Xu et al., 1995; Elia et al., 1999). It enables precise control of trace element concentrations, ensuring continuous exposure of roots to fortified nutrient solutions without soil interference. Furthermore, soilless cultivation can maximize the absorption, transport and accumulation of nutrients in the edible part of plant roots (Rouphael and Kyriacou, 2018). The utilization of soilless culture also facilitates the mitigation of soil-borne diseases and the optimization of plant nutrition (Gebreegziher, 2023).

Micro/nanobubble generation technology, developed at the end of the last century, has garnered increasing attention as a clean and efficient method for water treatment (Khuntia et al., 2012). Microbubbles are referred as bubbles with a diameter less than 100 microns and situated between micron- and nanoscale dimensions (Chang et al., 2024). They can generate through various mechanisms, such as rotational shear, pressure dissolution, electrochemistry, micropore pressure and mixed jet flow (Díaz-López et al., 2022). These bubbles possessed unique physical and chemical properties, including slow bubble rise in solution, prolonged residence time, enhanced solubility, expanded specific surface area, accelerated gas−liquid mass transfer rate (Wu et al., 2019), elevated interface point, and spontaneous generation of free radicals (Li et al., 2009). Recently, ozone micro/nanobubbles have emerged as a promising disinfection technology, facilitating prolonged aqueous-phase ozone reactivity. This innovative approach holds potential for preserving the freshness and quality of fruits and vegetables while safeguarding their overall nutritional value (Botondi et al., 2021). Moreover, it can also increase the ozone saturation concentration in water, leading to its decomposition into oxygen for crop growth and development, thereby mitigating hypoxia-related issues in crops (Fan et al., 2020).

Lettuce (*Lactuca sativa* L.) is one of the most commonly consumed leafy vegetables worldwide and widely used for soilless cultivation (Nerlich and Dannehl, 2021). Currently, hypoxia is primarily mitigated in crop soilless cultivation via water circulation by a pump to ensure continuous nutrient solution flow and enhance dissolved oxygen levels through the synergistic effects of water flow and air contact. However, the efficiency in large-scale hydroponic systems is relatively low, which poses challenges to the normal growth of plants. Therefore, it is necessary to develop new methods for irrigating crops and improving the oxygen content in water. The integration of soilless culture substrate and micro/nanobubble generation technology enables efficient and rapid dissolution of ozone into the nutrient solution, thereby augmenting the dissolved oxygen levels in the solution. In this study, lettuce plants were cultivated in a soilless substrate and irrigated with Yamazaki nutrient solution, micro/nano-ozone nutrient solution or micro/nano-oxygen nutrient solution (CK, O_2_ and O_3_). The effects on the physiological indices and rhizosphere microbial community diversity of lettuce were analyzed, providing a reference and theoretical basis for developing sustainable and green technology and can be a promising alternative to conventional methods for improving crop yields.

## 2 Materials and methods

### 2.1 Preparation of three types of nutrient solutions

(1) Japan Yamazaki lettuce nutrient solution (CK): The mother liquid of the nutrient solution was prepared according to the formula listed in **Supplementary Table S1**, mixed in a ratio of liquid A:B:C of 1:1:0.1 and diluted 100 times before use.

(2) Micro/nano oxygen bubble nutrient solution (O_2_): Air was compressed with an oxygen concentrator 8F-5AW device (Jiangsu Yuyue Medical Equipment Co., Ltd.), resulting in oxygen generation. Subsequently, the oxygen was conveyed through an intake pipe to a micro/nanobubble generator MF-5000 device (Shanghai Xingheng Technology Co., Ltd.) where it was allowed to interact with the CK nutrient solution for 20 minutes, forming a micro/nanooxygen bubble nutrient solution.

(3) Micro/nano-ozone bubble nutrient solution (O_3_): Air was compressed in the oxygen generator, resulting in oxygen generation. Subsequently, the produced oxygen was fed into the ozone generator VMUS-4 (Weihua Dike Beijing Science and Technology Co., Ltd.) for conversion into ozone. The concentration of gaseous-phase ozone could be determined using an online UV-300H ultraviolet ozone detector (Weihua Dike Beijing Science and Technology Co., Ltd.) and the OZONE DESTRUCT 30168-02 system (Weihua Dike Beijing Science and Technology Co., Ltd.). The generated ozone was then conveyed to the micro/nanobubble generator MF-5000 (Shanghai Xingheng Technology Co., Ltd.) through an intake pipe and allowed to interact with the CK nutrient solution for 20 minutes, thereby forming a micro/nano-ozone bubble nutrient solution. The ozone concentration in the water was measured using a GreenPrima PM8200CL ozone analyzer (GreenPrima Instrument Co., Ltd., Shanghai), which consists of a Bsens650 double platinum electrode sensor, a PM8200CL controller, and a BAF615 constant current trough.

### 2.2 Lettuce treatment and physical index measurements

Seeds of the Italian lettuce variety were obtained from China Vegetable Seed Technology Co., Ltd. (Beijing) and cultivated in a 4×8 seedling tray at 25 °C within a controlled greenhouse environment. Once the third leaf emerged, the seedlings were transplanted into Danish peat soil (PINDSTRUP 0-10 mm) substrate. The 36 lettuce plants with identical growth statuses were randomly divided into three groups. The plants were irrigated with 300 mL of micro/nano-ozone bubble nutrient solution (O_3_, ~30 mg/L), micro/nano-oxygen bubble nutrient solution (O_2_, ~30 mg/L), or Japan Yamazaki lettuce nutrient solution (CK) every two days. During the treatment, the temperature was maintained at 25 °C, and the pH of the nutrient solution was maintained at 6.2. The lettuce plants were harvested after 55 days, and the following physiological indicators were assessed. (1) The net photosynthetic rate, transpiration rate, conductance to H_2_O and intercellular carbon dioxide concentration of the lettuce plants were measured by an LI-6400XT portable photosynthesometer (Beijing Ecotek Technology Co., Ltd.) following the manufacturer’s instructions. (2) The rapid induction kinetic curve of chlorophyll fluorescence (OJIP curve) in lettuce leaves was assessed using a Handy-PE portable plant efficiency analyzer (Hansha Scientific Instruments Ltd.) to determine the chlorophyll fluorescence parameters. (3) The lettuce fresh weight was determined using a balance, followed by oven drying at 105 °C for 30 minutes. Subsequently, the dried plants were further subjected to drying at 80 °C for 48 hours before their dry weight was measured. (4) The nitrate content was determined according to the methods described by Wang et al. (2017a). The ascorbic acid content was determined using the 2,6-dichloro-isophenol method, while root activity was assessed using 2, 3, 5-triphenyltetrazolium chloride method (TTC) TTC method. (5) The plant height and spreading width of the lettuce plants were measured by a ruler, and the number of leaves was recorded.

### 2.3 Analysis of substrate physical and chemical properties

The rhizosphere substrate samples were collected after lettuce harvesting. A five-point sampling method was employed, and the substrates were gathered within a radius of 0-15 cm surrounding the root system (Zhao et al., 2024). Part of the samples were air-dried and used to determine physicochemical properties and enzyme activities. The remaining samples were immediately cryopreserved with liquid nitrogen and maintained at a temperature of -80 °C for amplicon sequencing. The samples included S, representing the original substrate; CK, representing the control group with nutrient solution irrigation; O_2_, representing the micro/nano oxygen bubble nutrient solution irrigation; and O_3_, representing the micro/nano ozone bubble nutrient solution irrigation. Each treatment consisted of 3 biological replicates.

Catalase (CAT) activity was measured with a Soil CAT Activity Assay Kit via spectrophotometry at a wavelength of 240 nm. The peroxidase (POD) activity was assessed by employing a soil-specific POD assay kit (Soil Peroxidase (S-POD) Activity Assay Kit, Spectrophotometer, Solarbio) using colorimetric analysis at a visible wavelength of 430 nm. The determination of urease activity (UE) was conducted using the Soil Urease (S-UE) Activity Assay Kit at a visible wavelength of 630 nm with a spectrophotometer. The sucrase (SC) activity was determined using a spectrophotometer with the Soil Saccharase (S-SC) Activity Assay Kit at a visible wavelength of 540 nm. The pH was determined using a pH meter (ST3100, Ohaus Instrument (Changzhou) Co., Ltd.). The soluble salt concentration (EC) was determined by a portable EC meter (TZS-ECW-G, Zhejiang Topu Yunnong Technology Co., Ltd.). The significant differences in all detection indicators were determined using ANOVA in R (v4.3.1), while pairwise *t* tests were used to assess the significant differences between pairwise samples.

### 2.4 DNA extraction, PCR amplification and sequencing

Total genomic DNA for each sample was extracted using the OMEGA Soil DNA Kit (M5635-02) (Omega Bio Tek, Norcross, GA, USA) following the manufacturer’s instructions and stored at -20 °C prior to further analysis. The quantity of the obtained DNA was measured using a NanoDrop NC2000 spectrophotometer (Thermo Fisher Scientific, Waltham, MA, USA) and agarose gel electrophoresis.

PCR amplification of the V3-V4 region of the bacterial 16S rRNA gene was performed using the forward primer 338F (5’-ACTCCTACGGGAGGCAGCA-3’) and reverse primer 806R (5’-GGACTACHVGGGTWTCTAAT-3’). The fungal primers ITS5F (5’-GGAAGTAAAAGTCGTAACAAGG-3’) and ITS1R (5’-GCTGCGTTCTTCATCGATGC-3’) were used to amplify the V1 region of the ITS gene. Sample-specific 7-bp barcodes were incorporated into the primers for multiplex sequencing. The PCR mixture contained 5 μL of reaction buffer (5×) and high GC buffer (5×), 0.25 μL of Q5 high-fidelity DNA polymerase (5 U/μL), 2 μL (10 mM) of dNTPs, 1 μL (10 µM) of each forward and reverse primer, 2 μL of DNA template, and 8.75 μL of ddH_2_O. Thermal cycling consisted of initial denaturation at 98 °C for 30 s, followed by 25~30 cycles of denaturation at 98 °C for 15 s, annealing at 50 °C for 30 s, and extension at 72 °C for 30 s, with a final extension of 5 min at 72 °C. PCR amplicons were purified with an Axygen Gel Extraction Kit (Axygen Biosciences, Union City, CA, USA) and quantified using a Quant-iT PicoGreen dsDNA Assay Kit (Invitrogen, Carlsbad, CA, USA) on a microplate reader (BioTek, FLx800). After the individual quantification step, amplicons were pooled in equal amounts, and paired-end 2×250 bp sequencing was performed using the Illumina NovaSeq platform with a NovaSeq 6000 SP Reagent Kit (500 cycles) at Shanghai Personal Biotechnology Co., Ltd. (Shanghai, China).

### 2.5 Quality control and analysis of sequence data

The QIIME2 (v2019.4) protocol was used with slight modifications according to the official tutorials (https://docs.qiime2.org/2019.4/tutorials/) to conduct data analysis (Bolyen et al., 2018). After removing the primers using the cutadapt plugin and filtering, denoising, merging, and removing chimeras from the raw sequences using the DADA2 plugin, the obtained high-quality sequences that exhibited 100% similarity were subsequently clustered into amplicon sequence variants (ASVs) (Callahan et al., 2016). For the annotation of bacterial 16S rDNA and ITS, we used the Greengenes (v13.8) (DeSantis et al., 2006) and UNITE databases (v8.0) (Kõljalg et al., 2013) to assign taxonomic information. ASVs with abundances less than 0.001% of the total samples were removed, and the abundance matrix with rare ASVs removed was used for subsequent analyses. We used the QIIME feature-table rarefy function for data normalized for differences in sequencing depth, and the leveling depth was set to 95% of the minimum sample sequence size. We conducted a comparative analysis of the bacterial and fungal proportions and percentages across various taxonomic levels (domain, kingdom, phylum, class, order, family and genus) within each sample to further investigate variations in the composition of the substrate microbiota.

### 2.6 Species diversity analysis

The α diversity indices, including Chao1, Shannon (Shannon, 1948), observed species, Simpson, Pielou’s evenness, and Goods’ coverage, were calculated using QIIME2 and visualized as box plots to compare the species richness and evenness of bacteria and fungi. Using R and QIIME2 software, β-diversity analysis was performed using the Bray-Curtis metric to investigate changes in microbial community structure between samples, which can be visualized by PCoA. The unweighted pair-group method with arithmetic means (UPGMA) hierarchical clustering of samples according to Euclidean distance based on species composition profiles at the genus level was used. To compare the memberships and structures of communities between samples, heatmaps were generated with the top 20 ASVs using Mothur.

### 2.7 Correlation analysis between physicochemical properties and microbial communities

Based on the physicochemical parameters and the relative abundances of the bacterial and fungal communities, redundancy analysis (RDA) was conducted using Canoco 4.5 software.

### 2.8 Analysis of key microbiota

To identify key bacteria and fungi within different groups, we employed linear discriminant analysis effect size (LEfSe) to analyze characteristic strains using the default parameters (Segata et al., 2011). The Kruskal-Wallis test was applied with a significance threshold of 0.05 for variance analysis. Similarly, the Wilcoxon rank sum test was conducted with a significance threshold of 0.05. Moreover, discriminating features were determined based on a logarithmic LDA score threshold of 3.0.

## 3 Results

### 3.1 Effects of different nutrient solutions on plant height, degree of development and yield in lettuce

**Figure 1:**
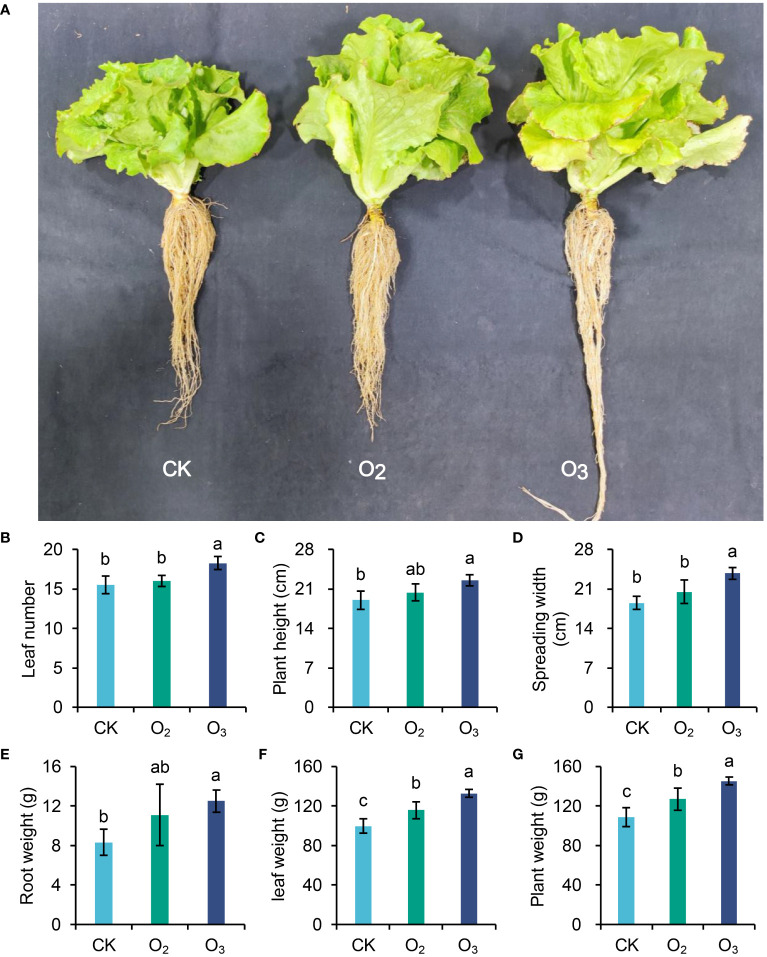
Effects of different irrigation patterns on plant height, spreading width and weight per lettuce plant. **(A)** Graph of the lettuce plants harvested from the three different groups. **(B–G)** Comparisons of leaf number, plant height, spreading width, root weight, leaf weight and whole plant weight between the three groups. The significance of differences was determined using ANOVA with pairwise *t* tests. Different letters on different columns indicate a significant difference between these two treatments (*p* < 0.05).

After irrigation with O_2_, O_3_ or CK nutrient solution, we measured the lettuce leaf count, plant height, and individual plant weight (**Figure 1**). Compared with the CK group, the O_3_ treatment significantly increased the number of lettuce leaves and promoted both the growth and development of the plants. Although the O_2_ group also exhibited some promotion effects on lettuce plants, these effects did not reach statistical significance (**Figures 1B–D**, *p* > 0.05). The application of micro/nanobubble nutrient solution for irrigating lettuce significantly increased the wight per plant (**Figures 1E–G**, *p <*0.01), with a more pronounced promoting effect observed in the O_3_ group than in the O_2_ group. Therefore, irrigation with micro/nanobubble nutrient solutions, especially those containing ozone, can effectively enhance lettuce production.

### 3.2 Effects of different nutrient solutions on root activity and ascorbic acid and nitrate contents of lettuce plants

There was a significant increase in the root vitality of the lettuce plants in the O_2_ and O_3_ groups compared to that in the CK group (*p* < 0.05); the root viability coefficient of the O_3_ group was significantly greater than that of the O_2_ group (**Table 1**). The ascorbic acid content of the lettuce plants was not significantly affected by the nutrient solutions. Compared with that in the CK group, the application of the micro/nanobubble nutrient solution resulted in a significant reduction in the nitrate content (*p* < 0.01), and the O_3_ group exhibited significantly lower nitrate levels than the O_2_ group. Therefore, during substrate cultivation, the application of micro/nanobubble generation technology to introduce oxygen or ozone into nutrient solution could effectively enhance root vitality and significantly mitigate nitrate accumulation in lettuce plants.

**Table 1:** Effects of microbubble technology in nutrient solution on the root activity, ascorbic acid content and nitrate content of lettuce plants.

Group	Root vigor/mg·g^-1^h^-1^	Ascorbic acid/mg·L^-1^	Nitrate/mg·kg^-1^
CK	0.66 ± 0.22c	0.122 ± 0.04a	3.42 ± 0.68a
O_2_	0.72 ± 0.23b	0.117 ± 0.04a	1.99 ± 0.35c
O_3_	1.03 ± 0.21a	0.152 ± 0.07a	2.93 ± 0.49b

The significance of differences was determined using ANOVA with pairwise *t* tests. Different letters on different columns indicate a significant difference between these two treatments (*p* < 0.05).

### 3.3 Effects of different nutrient solutions on the photosynthetic parameters of the lettuce plants

Compared with that of the CK group, the net photosynthetic rate of the O_2_ group exhibited a slight increase but lacked statistical significance; however, the net photosynthetic rate of the O_3_ group significantly improved (**Table 2**). Moreover, both the O_2_ and O_3_ groups exhibited significantly increased transpiration rates, intercellular carbon dioxide (CO_2_) concentrations, and conductance to H_2_O (*p* < 0.05). Furthermore, the net photosynthetic rate, transpiration rate, intercellular carbon dioxide concentration, and conductance to H_2_O in the O_3_ group were significantly greater than those in the O_2_ group (*p* < 0.05). These results suggested that incorporating micro/nanobubble generation technology into soilless substrate cultivation processes was beneficial for enhancing leaf conductance to H_2_O, increasing the intercellular CO_2_ concentration, and substantially improving the lettuce transpiration rate.

**Table 2:** Effects of different nutrient solutions on the photosynthetic parameters of lettuce plants.

Group	Net photosynthetic rate (μmol·m^-2^·s^-1^)	Transpiration rate (mmol·m^-2^·s^-1^)	Intercellular CO_2_ concentration (μmol·mol^-1^)	Conductance to H_2_O (mol·m^-2^·s^-1^)
CK	11.12 ± 1.41b	1.00 ± 0.07c	128.73 ± 47.87c	0.05 ± 0.00c
O_2_	11.38 ± 1.61b	1.82 ± 0.03b	220.63 ± 27.29b	0.09 ± 0.00b
O_3_	13.32 ± 2.36a	2.27 ± 0.02a	238.33 ± 32.69a	0.12 ± 0.00a

Different letters on different columns indicate a significant difference between these two treatments (*p* < 0.05).

### 3.4 Effect of micro/nanobubble nutrient solutions on the chlorophyll fluorescence parameters of lettuce plants

**Figure 2:**
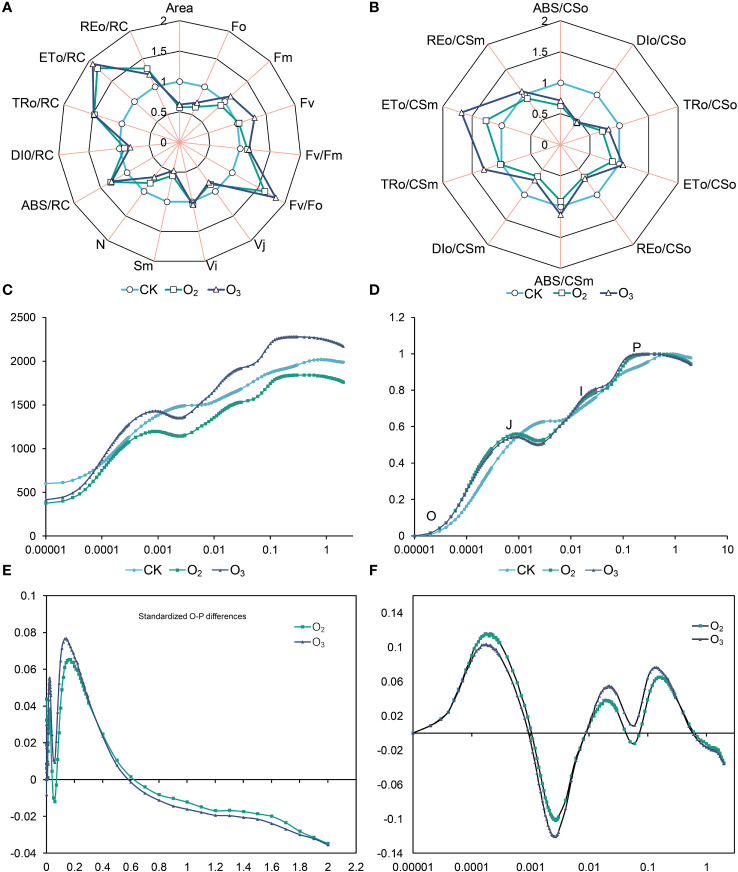
Effects of different micro/nanobubble nutrient solutions on the chlorophyll fluorescence parameters of lettuce leaves. **(A, B)** The electron transport and reaction center activity of the PSII receptor side of lettuce leaves. **(C)** OJIP curves of lettuce leaves in the CK, O_2_ and O_3_ groups. **(D)** Fluorescence O-P logarithmic curve. **(E)** OJIP curves of the fluorescence kinetics of the O_2_ and O_3_ groups after global (O~P) double standardization with the CK group. **(F)** Logarithmic OJIP curves of the fluorescence kinetics of the O_2_ and O_3_ groups after global (O~P) double standardization with the CK group.

Compared to those of the CK group, the S_m_, V_j_, DI_o_/CS_o_ and DI_o_/CS_m_ values of the O_3_ and O_2_ groups decreased significantly; the potential photochemical activity (F_v_/F_o_), maximum photochemical efficiency (F_v_/F_m_), ABS/RC, TR_o_/RC, RE_o_/RC, ET_o_/RC and ET_o_/CS_o_ increased, respectively (**Figures 2A, B**). Our results indicated that irrigating lettuce with nutrient solution after micro/nano treatment enhances the efficiency of chlorophyll absorption and light energy transfer, especially the O_3_ group.

As shown in **Figure 2C**, compared with that of the CK group, the initial fluorescence intensity (F_o_) of the O_3_ and O_2_ groups decreased by 29% and 37%, respectively; the O_3_ group exhibited a 13% increase in the maximum fluorescence intensity (F_m_), while the O_2_ group showed a 9% decrease. Additionally, when the PSII reaction centers were completely closed, the O_3_ group demonstrated a greater fluorescence yield than the O_2_ group did. Compared to the CK group, the fluorescence intensity of the J-spot was attenuated, while that of the I-spot and P-spot were enhanced. O_3_ irrigation resulted in a reduction in the fluorescence intensity of the J-spot, I-spot and P-spot. After standardizing the OJIP curve (**Figure 2D**), it was observed that the fluorescence intensity of the J and P points in the O_3_ and O_2_ groups was significantly greater than that the CK group. The results suggested that O_3_ and O_2_ groups can enhance electron transfer in both the PSII reaction center and acceptor side. Notably, significant changes were observed at points J (2 ms) and I (30 ms) (**Figures 2E, F**). Overall, the effects of O_2_ and O_3_ treatment on the OJIP curve of lettuce leaves were comparable, while the impact of O_3_ on the oxygen emission complex (OEC) of lettuce leaves exceeded that of O_2_.

### 3.5 Analysis of physical and chemical properties of the substrate

**Figure 3:**
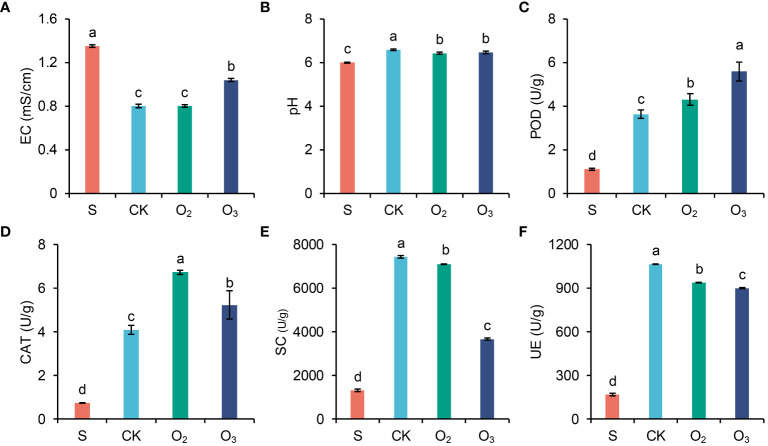
Analysis of the physical and chemical properties of the substrate. Values of EC **(A)**, pH **(B)**, POD **(C)**, CAT **(D)**, SC **(E)** and UE **(F)** between the three groups. The significant differences were determined using ANOVA with pairwise t tests.

**Figure 3** shows that the indices of pH, CAT activity, POD activity, UE activity, and SC activity increased across all the three groups irrigating with nutrient solution (O_3_, O_2_ and CK) compared to those of the S group. Additionally, a decrease in EC was observed compared to that in the S group. Notably, the EC of the O_3_ group exhibited a slight increase compared to that of both the CK and O_2_ groups (**Figure 3A**). Compared to those of the CK group, both the O_3_ and O_2_ groups exhibited a decrease in pH (**Figure 3B**). POD enzyme activity significantly increased, with the greatest increase observed in the O_3_ group (**Figure 3C**). CAT activity increased more in the O_2_ group than in the O_3_ group (**Figure 3D**). SC and UE activities were lower in both the O_2_ and O_3_ groups than the CK group, with a particularly significant decrease observed in SC activity within the O_3_ group (**Figures 3E, F**).

### 3.6 Microbial sequencing analysis

**Figure 4:**
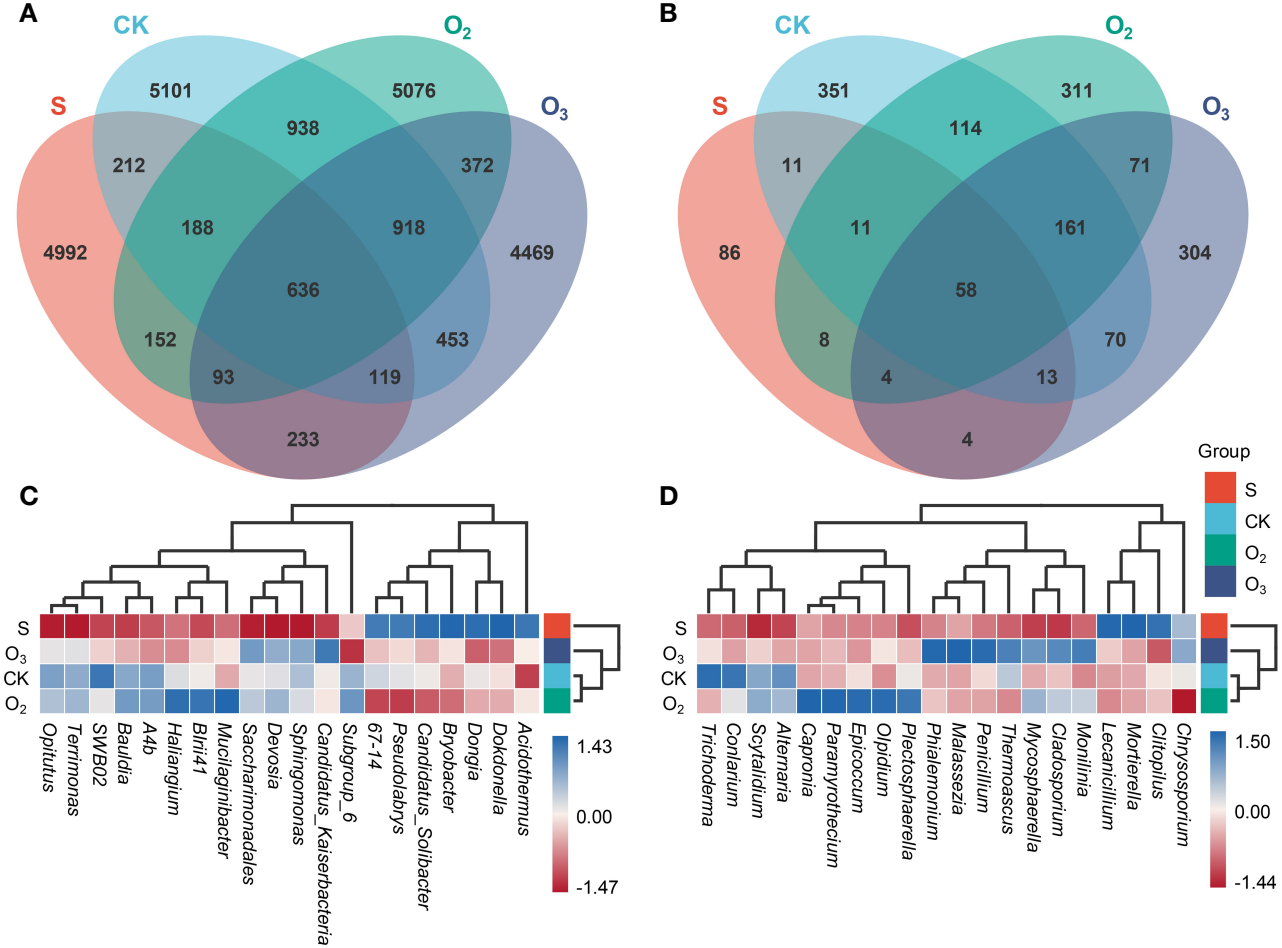
ASV comparison of microbial communities and the abundance profile of the top 20 genera between different groups. **(A, B)** Vann comparisons of ASVs identified among the bacterial and fungal communities. **(C, D)** The abundance profiles of the top 20 genera among four samples, and UPGMA clustering of the samples was conducted according to the Euclidean distance of the species composition data.

Totally, 12 libraries were sequenced, which obtained 1,663,178 and 907,842 raw bacterial and fungal sequences. After quality control (QC) and data filtering, 1,500,641 and 828,149 clean reads were obtained for analysis of bacterial and fungal communities (**Supplementary Table S2**). In the bacterial community, the numbers of ASVs in the S, CK, O_2_ and O_3_ groups were 6,625, 8,565, 8,373 and 7,293, respectively. The number of common ASVs across all four groups was 636, accounting for 9.6%, 7.43%, 7.60% and 8.72% of the total number of ASVs identified in each group, respectively (**Figure 4A**). The fungal community contained 195 ASVs in the S group, while those in the CK, O_2_ and O_3_ groups numbered 789, 738 and 685, respectively. There were 58 shared ASVs among these four groups, accounting for 29.74%, 7.35%, 7.86% and 8.47% of the total number of ASVs identified in each corresponding group, respectively (**Figure 4B**). Overall, the ASV increased in the substrate after lettuce cultivation and irrigation, but there was a significant difference in composition among the three groups. The total number of ASVs in both groups treated with O_2_ and O_3_ was lower than that in the CK group, indicating a reduced survival ability for microorganisms in these environments. Especially, the O_3_ group had a more noticeable impact on the total number of ASVs.

Clustering analysis based on the genus-level abundance of different classes in the microbial communities revealed that Acidothermus, Dokdonella, Dongia, Bryobacter, Candidatus_Solibacter, Pseudolabrys and 67-14 exhibited relatively greater abundances in the S group. Conversely, Subgroup 6, Candidatus_Kaiserbacteria, Sphingomonas, Devosia, Saccharimonadales, Mucilaginibacter, BIrii41, Haliangium, A4b, Bauldia, SWB02, Terrimonas and Opitutus displayed lower abundances than they did in the other three groups (CK, O_2_ and O_3_) (**Figure 4C**). The species compositions of the CK and O_2_ groups exhibited a relatively high degree of similarity. In terms of the fungal community, significant differences were observed in the distribution of the dominant bacteria, with Chrysosporium, Clitopilus, Mortierella, and Lecanicillium being relatively abundant in the S group. Conversely, Chrysosporium, Plectosphaerella, Olpidium, Epicoccum, Paramyrothecium, Capronia, Alternaria and Scytalidium were relatively highly abundant in the O_2_ group. Monilinia, Cladosporium, Mycosphaerella, Thermoascus, Penicillium, Malassezia and Phialemonium were relatively abundant in the O_3_ group. Additionally, the relative abundances of Alternaria, Scytalidium, Conlarium, and Trichoderma were greater in the CK group (**Figure 4D**). These results demonstrated that the relative abundance of the dominant bacteria was influenced by treatment with the micro/nanobubble nutrient solutions.

### 3.7 Alpha diversity of the microbial community

**Figure 5:**
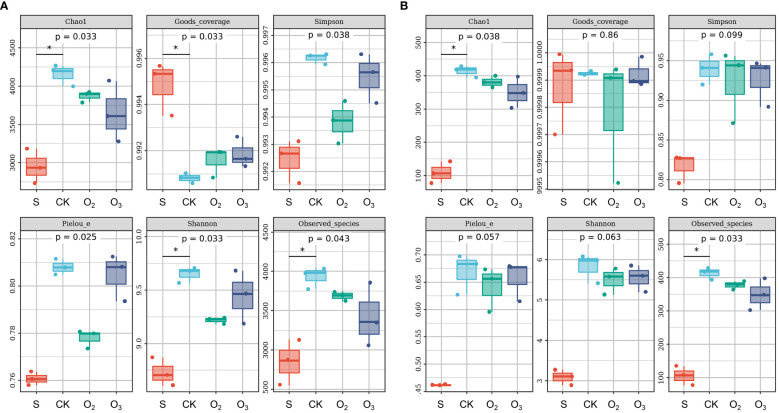
The α diversity of the microbial community among the different groups. **(A)** The α diversity of the bacterial community. **(B)** The α diversity of the fungal community, including Chao1, Good’s coverage, Simpson, Pielou’s evenness, Shannon and observed species indices. * indicate significant differences among the different groups (Dunn’s test, *p* < 0.05).

Compared with those of the CK group, the α diversity of S group exhibited lower Chao1, Simpson, Pielou’s evenness, Shannon, and observed species indices in the bacterial community (**Figure 5A**). Conversely, the CK group displayed higher values than the O_2_ and O_3_ groups. These findings suggested that lettuce cultivation enhanced bacterial diversity and that micro/nanobubble nutrient solution decreased this diversity. However, no significant difference was detected in terms of the α diversity (**Figure 5B**, *p* > 0.05). That the rarefaction curves tended to be flat indicated that the sequencing results effectively reflected the diversity present in the sample (**Supplementary Figures S1A, B**). The bacterial abundance curve exhibited a relatively gentle slope (**Supplementary Figure S1C**), indicating small differences in abundance between the ASVs and high uniformity in community composition. In contrast, the fungal community displayed a steep line (**Supplementary Figure S1D**), suggesting low uniformity in community composition. Hence, both the abundance and diversity of bacterial and fungal communities in the substrate increased after plant cultivation and decreased following treatment with micro/nanobubble nutrient solutions.

### 3.8 Analysis of the microbial community structure composition

**Figure 6:**
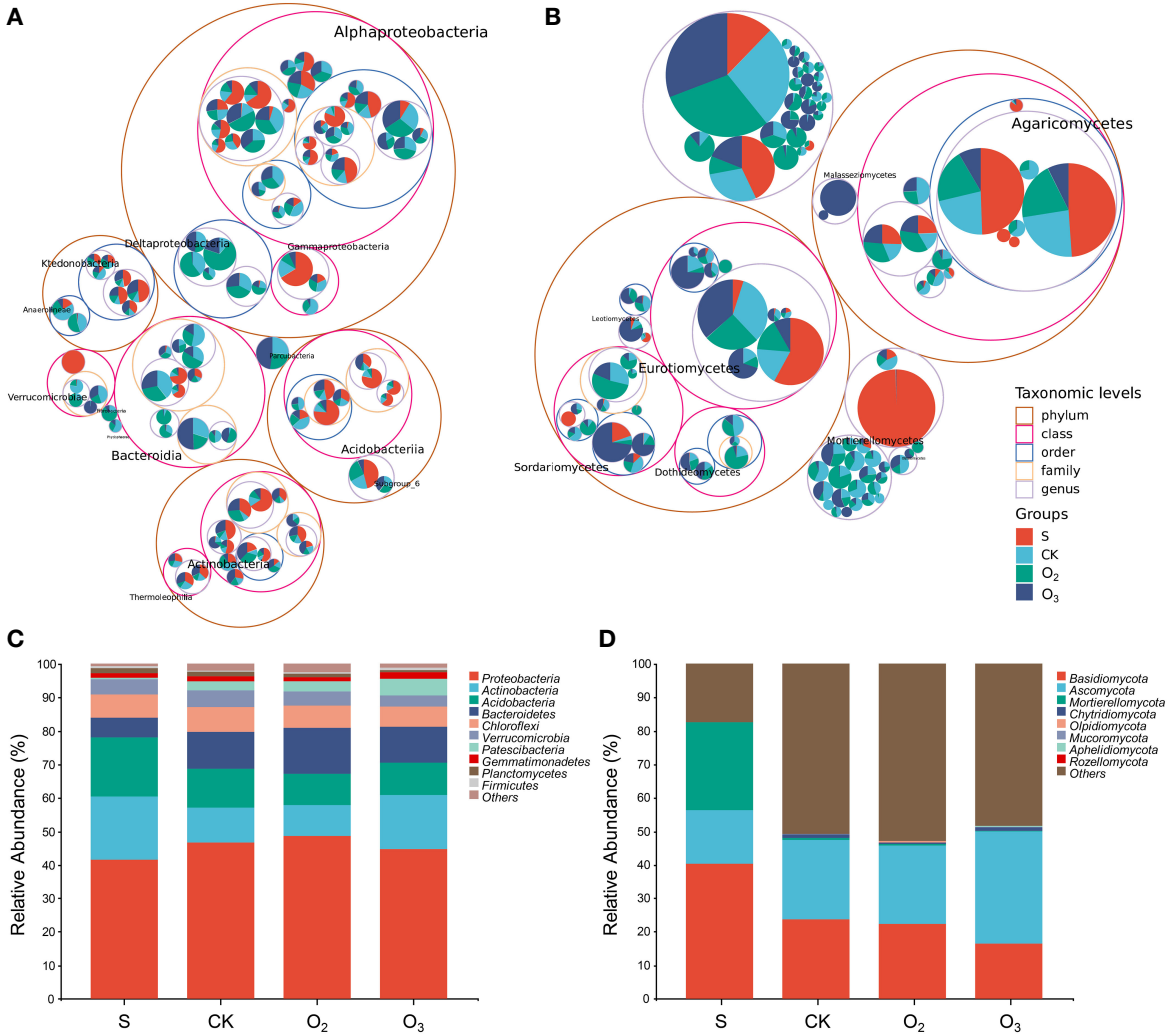
Classification tree and taxonomic composition of the microbial community structures among the different groups. **(A, B)** The classification tree of the detected bacteria and fungi. **(C, D)** The taxonomic composition of bacterial and fungal community structures at the phylum level.

To investigate the impact of micro/nanobubble nutrient solutions on the dominant bacterial community structure and fungi, we analyzed and compared the species composition of each group. **Figure 6A** showed the classification tree of the bacterial communities in the four substrate samples. There were mostly four phyla that were the most abundant, namely, Proteobacteria, Actinobacteria, Acidobacteria, and Chloroflexi. Among them, Alphaproteobacteria, Actinobacteria, Acidobacteriia, and Ktedonobacteria belong to these phyla. Moreover, two classes, Bacteroidia and Verrucomicrobiae, also showed high abundance. **Figure 6B** showed the classification tree of the fungal community, which mostly included two phyla, Ascomycota and Basidiomycota. Among them, Eurotiomycetes, Sordariomycetes, Dothideomycetes and Agaricomycetes had the highest abundances.

Proteobacteria consistently dominated the bacterial communities across the three treatment groups (**Figure 6C**, CK, O_2_ and O_3_); however, variations in relative abundance were observed. The phyla Proteobacteria, Actinobacteria, Acidobacteria, Bacteroidetes, and Chloroflexi collectively accounted for 88% of the bacterial composition among all groups. Conversely, Verrucomicrobia, Patescibacteria, Gemmatimonadetes, Planctomycetes, and Firmicutes exhibited relative abundances below 5%. Compared to that in the CK group, the relative abundance of Acidobacteria decreased in both the O_2_ and O_3_ groups (**Figure 6C**). In the O_2_ group, there was a decrease in the proportion of Actinobacteria, while the relative abundances of Proteobacteria and Bacteroidetes increased. In the O_3_ group, there was a decreasing trend in the relative abundances of Proteobacteria and Bacteroidetes, while the proportions of Actinobacteria and Patescibacteria increased.

The composition of the fungal community was relatively simple compared to that of the bacterial community (**Figure 6D**). In terms of relative abundance, the top 8 phyla of samples included Basidiomycota, Ascomycota, Mortierellomycota, Chytridiomycota, Olpidiomycota, Mucoromycota, Aphelidiomycota, and Rozellomycota. Compared with those in the S group, the relative abundance of Basidiomycota and Mortierellomycota decreased, and that of Ascomycota increased in the substrate (CK, O_2_ and O_3_). Compared with than in the CK group, the relative abundance of Mortierellomycota in the O_2_ and O_3_ groups significantly decreased. Moreover, there was a decreasing trend in the relative abundance ratio of Basidiomycota and Chytridiomycota, while an increase was noted in the relative abundance ratio of Olpidiomycota. Compared to those in the CK group, the relative abundances of Ascomycota and Mucoromycota in the O_2_ group decreased. Conversely, in the O_3_ group, there was an increase in the relative abundance of Ascomycota, Mucoromycota, and Aphelidiomycota.

**Figure 7:**
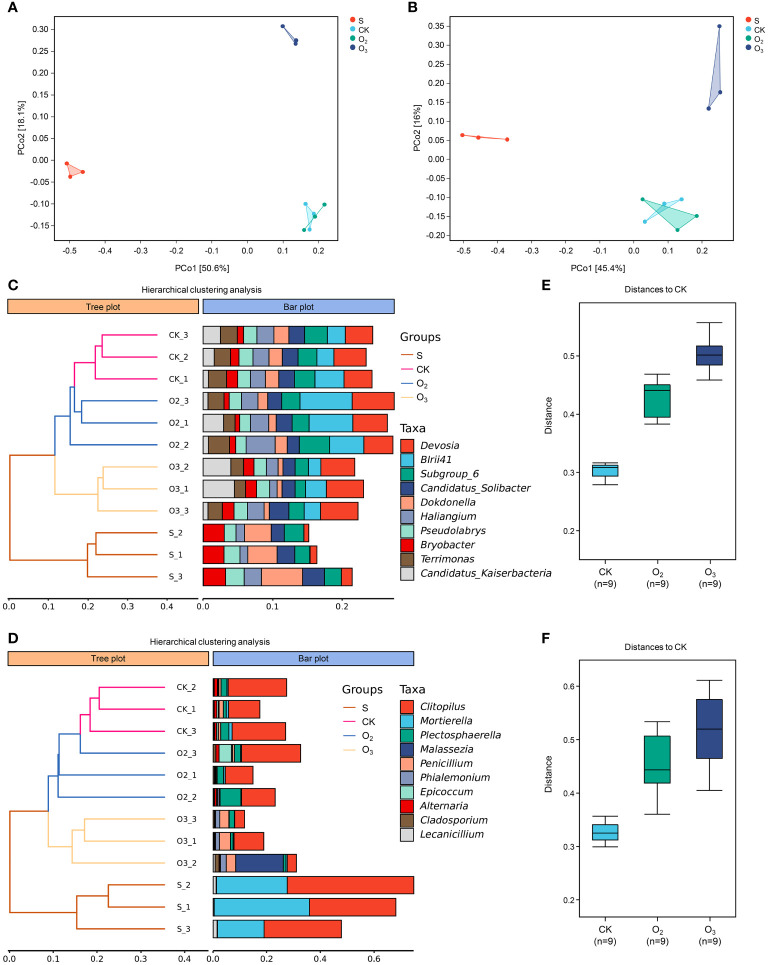
β diversity analysis of the microbial community structures. **(A, B)** PCoA plots of bacterial and fungal communities. **(C, D)** The panel on the left is a hierarchical clustering diagram in which samples are clustered according to their similarity of bacterial and fungal communities. The panel on the right is a stacked bar chart of the top 10 most abundant genera. **(E, F)** Analysis of the distance from O_2_ and O_3_ to CK based on their microbial abundance profiles.

Principal Coordinate Analysis (PCoA) is a conventional unconstrained ranking technique that can be employed to depict variations or associations in the internal composition of microbial communities. As shown in **Figure 7A**, principal component 1 (PCo1) accounted for 50.6% of the bacterial community diversity within the samples, while PCo2 explained an additional 18.1%. When considering fungal community diversity (**Figure 7B**), PCo1 and PCo2 jointly contributed to approximately 61.4% of the observed variation. **Figures 7C, D** show that all three biological replicates from each treatment, except the O_2_ treatment, were clustered into one cluster at the genus level (the same was also true at the other levels), indicating that the micro/nanobubble nutrient solutions changed the structural distribution of bacteria and fungi in the substrate. The topological structure of the hierarchical clustering of bacteria and fungi exhibited remarkable similarity, with both the CK and O_2_ groups closely clustered together. The S group was positioned at the base of the topology, indicating that lettuce cultivation most significantly influenced substrate microorganisms. Intergroup difference analysis revealed that the distance among the three sampling points in the CK group was the smallest, and that the distance between CK and O_2_ or CK and O_3_ was much greater (**Figures 7E, F**). This further confirmed that the effects of the intergroup treatment outweighed the effects of the sampling deviations. Totally, these results indicated that the microbial and fungal communities in the rhizosphere substrate changed after treatment with two micro/nanobubble nutrient solutions and that the community composition was more influenced by O_3_.

### 3.9 Principal coordinate analysis and redundancy analysis

**Figure 8:**
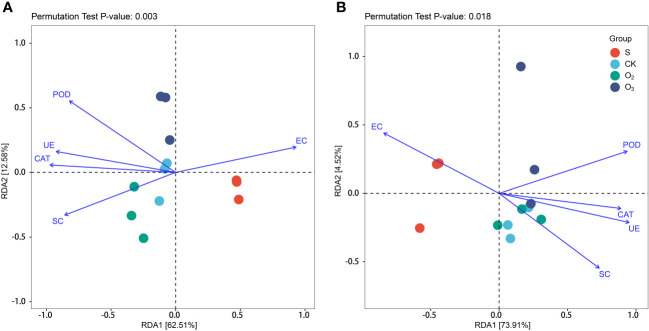
RDA plots of microbial communities with environmental variables. **(A)** Bacterial communities. **(B)** Fungal communities. POD, CAT, UE, SC and EC.

To further investigate the impact of environmental factors on the distribution of substrate microbial community components, such as CAT, POD, UE, SC, pH and EC, RDA was employed to explore the relationships between the composition and structure of the substrate microbial community and environmental variables. As shown in **Figure 8A**, it shows that the significant differences of the bacterial community structure between the S, CK, O_2_ and O_3_ groups were correlated with the environmental conditions. RDA1 accounted for 62.51% of the interpretive variance, while RDA2 explained an additional 12.58%. Together, these two components contributed to a cumulative interpretive degree of 75.09%, indicating a high level of explanatory power. The RDA of fungal communities revealed that the incorporation of environmental factors and other variables led to more pronounced dissimilarities in the community structure composition between the S, CK, O_2_ and O_3_ groups. The first component accounted for 73.91% and the second component 4.52% (**Figure 8B**). The CAT, POD, UE, SC and pH were found to be important environmental factors affecting both the bacterial and fungal community. For the bacterial community (**Figure 8A**), the S group was positively correlated with EC; the CK group positively correlated with pH, UE and SC, the O_2_ group exhibited a positive correlation with SC; and the O_3_ group displayed a positive correlation with CAT and POD. For the fungal communities (**Figure 8B**), the S group was positively correlated with EC; the CK group showed a positive correlation with SC; the O_2_ group exhibited a positive correlation with pH, UE and SC; and the O_3_ group displayed a positive correlation with SC, CAT, and POD.

### 3.10 LEfSe difference analysis

**Figure 9:**
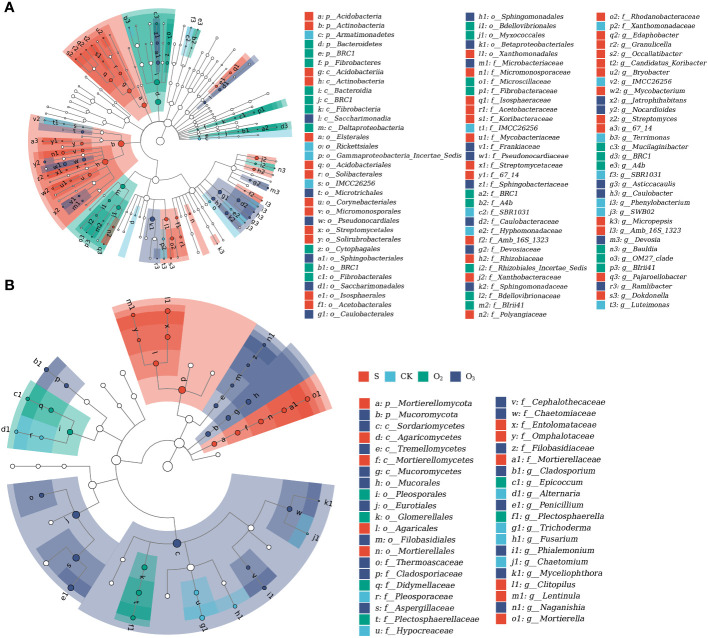
LEfSe analysis of microbial sequences. **(A)** Bacterial 16S rDNA sequences. **(B)** Fungal ITS rDNA sequences. The concentric circles depict the hierarchical classification levels ranging from phylum to genus. Each small circle at a specific level represents a taxonomic classification, with its size directly proportional to the relative abundance of that taxon. Hollow nodes represent taxa with no significant differences between groups, while colored nodes indicate significant differences between groups S, CK, O_2_ and O_3_, with higher abundances in samples represented by these colors. Letters were used to identify taxa that exhibited significant differences between groups. The legend on the right provides the names of the species denoted by letters. The size of each node corresponds to the average relative abundance of the taxon.

LEfSe analysis was employed to identify biomarkers in microbial communities exhibiting significant differences among the groups. For the bacterial communities, 38, 14, 25, and 21 biomarkers were characterized for the S, CK, O_2_ and O_3_ groups, respectively (**Figure 9A**). Actinobacteria exhibited the greatest difference in abundance in the S group, whereas Terrimonas and Deltaproteobacteria were identified as the most abundant bacterial taxa in the CK and O_2_ groups, respectively. Devosia emerged as the predominant taxon in the O_3_ group. In the fungal community, there were 11, 6, 6, and 18 biomarkers in S, CK, O_2_ and O_3,_ respectively (**Figure 9B**). Among these groups, Clitopilus was identified as the most significant fungus in the S group, Fusarium exhibited the highest abundance in the CK group, Plectosphaerella showed significant enrichment in the O_2_ group, and Sordariomycetes emerged as the most significant fungus in the O_3_ group.

## 4 Discussion

Recently, the use of ozone water in agricultural production has significantly increased, including its application in the preservation of agricultural products (Shi et al., 2023), enhancement of fruit quality (Silva Neto et al., 2019), sterilization and disinfection, and disease prevention and control (Trinetta et al., 2011). By incorporating micro/nanobubble generation technology into agricultural practices, it not only conserves agricultural water but also enhances crop quality and yield (Liu et al., 2019). Due to the historical significance and economic value as a vital vegetable crop, employing micro/nanobubble generation technology alongside soilless substrate culture has immense potential for optimizing lettuce cultivation systems and increasing productivity.

### 4.1 Effects of micro/nano-ozone bubble nutrient solution on the growth, quality, and photosynthetic parameters of lettuce

Improving the lettuce quality and yield is a major concern in lettuce cultivation. Yang et al. (2017) reported that the use of glutamine as a nitrogen source in hydroponic cultivation can increase the accumulation of glycosylated flavonoids, ascorbic acid, and most amino acids, and improve the nutritional value of lettuces. Foliar application of selenium solution for lettuce plants can enhance their nitrogen utilization efficiency (Piñero et al., 2022). It has also been shown that irrigation with oxygenated water can increase crop yield (Zhou et al., 2019). In this study, compared with the CK group, the O_2_ and O_3_ treatments increased the lettuce yield (**Figure 1G**) and improved the root system vitality (**Table 1**). This treatment may facilitate the promotion of aerobic respiration in plant roots, thereby augment nutrient transfer (Sey et al., 2009; Liu et al., 2019), which can stimulate the growth of lettuce plants.

Ascorbic acid is abundant in fresh vegetables and fruits (Zheng et al., 2022), with lettuce being considered a moderate dietary source of this essential nutrient (Yang et al., 2021). In this study, the content of ascorbic acid in the O_3_ group was greater than the CK group. Yao et al. (2021) demonstrated that the utilization of micro/nano oxygen aeration technology can enhance the oxidation efficiency of ammonia and effectively eliminate NH_4_^+^ through improved oxygen transfer efficiency. Similar to Yao et al. (2021), the nitrate content in the lettuce leaves of both O_2_ and O_3_ groups decreased compared to the CK group, especially the O_2_ group (**Table 1**).

It has demonstrated that the introduction of oxygen can enhance crop yield, water use efficiency, and the leaf photosynthetic rate (Zhou et al., 2019). The net photosynthetic rate, transpiration rate, intercellular carbon dioxide concentration, and stomatal development in the O_3_ group were significantly greater than those in the O_2_ group (**Table 2**). When a nutrient solution treated with micro/nanobubble generation technology is applied for lettuce irrigation, it can improve the net photosynthetic rate, and promote the efficient absorption and transfer of light energy, thereby promote lettuce growth and development. In conclusion, the micro/nanobubble nutrient solution not only promotes lettuce growth but also enhances its quality, especially the O_3_ treatment.

### 4.2 Effect of micro/nano-ozone bubble nutrient solution on microbial community diversity in the substrate

The exceptional disinfectant and antibacterial properties of ozone stem from its potent oxidizing effect. It has demonstrated bactericidal activity against both gram-positive and gram-negative bacteria, in addition to its ability to effectively deactivate yeast and mold spores (Wang et al., 2021). Ozone micro/nanobubbles can effectively remove the antibiotics ciprofloxacin and levofloxacin from water (Babaee et al., 2023). Dissolved ozone exhibits greater antibacterial efficacy than ozone in its gaseous state and can effectively control bacteria, fungi, viruses, protozoa, and spores by impacting cellular membranes and other cellular constituents (Premjit et al., 2022; Shelake et al., 2022).

Ozone water irrigation may induce denaturation and deactivation of harmful substances within the plant rhizosphere, thereby enhancing plant growth. There was a significant increase in both the abundance and diversity of bacterial and fungal communities present in the substrate (**Figure 5**). The species number and diversity of bacteria and fungi in the substrates of the O_2_ groups were slightly lower than the CK group, while those in the O_3_ group were significantly lower. This may be due to ozone’s potent oxidation and antibacterial activity. Microbial community analysis revealed similar dominant phyla of bacteria and fungi among the sampled substrates; however, their relative abundances varied. Actinobacterium is pivotal in the decomposition of cellulose and various chemical compounds, thereby significantly contributing to the carbon cycle (Lewin et al., 2016). The relative abundance of Actinobacteria in both the CK and O_2_ groups exhibited low, whereas that in the O_3_ and S groups showed high. Bacteroidetes are the predominant bacteria in soil (Larsbrink and McKee, 2020) and can secrete a diverse array of carbohydrate-active enzymes. Compared to those in the S group, the relative abundances of Bacteroidetes in the CK, O_2_ and O_3_ groups increased. The abundance of Bacteroidetes in the O_3_ group was lower than that in the O_2_ group but was consistent with that in the CK group (**Figure 6C**). Previous studies have shown that bacteria of the Acidobacteria phylum are oligotrophic (Wang et al., 2017b) and that the abundance of Acidobacteria in soil is often greater under lower organic carbon conditions (Fierer et al., 2007). The relative abundance of Acidobacteria in the substrates of groups O_2_ and O_3_ decreased. Proteobacteria are copiotrophically attributed and favored by nutrient-rich conditions with high carbon content (Fierer et al., 2007; Newton and McMahon, 2010). In all the groups, more than 40% of the bacteria belonged to Proteobacteria. Chloroflexi is an anaerobic bacterium that undergoes parthenogenetic reproduction and primarily engages in carbon and nitrogen fixation (Logue and Lindström, 2010). The relative abundance of Chloroflexi remained consistent across all groups. The findings suggested that the introduction of oxygenated irrigation did not impact the population of beneficial bacteria but did positively influence nutrient accumulation in the substrate.

The relative abundance of Basidiomycota decreased in the CK, O_2_ and O_3_ groups compared to that in the S group, while there was a significant decrease in the relative abundance of Mortierellomycota and an increase in that of Ascomycota. The Basidiomycota phylum can be broadly categorized into saprophytic, symbiotic, and parasitic/pathogenic taxa based on their nutritional strategies. Saprophytic fungi can decompose organic matter such as wood, leaf litter or feces, while symbiotic fungi establish mutualistic associations with other organisms. Therefore, these fungi are pivotal in ecosystem functioning. Pathogenic or parasitic fungi can infect plants and other fungi (Schmidt-Dannert, 2016). The Mortierella fungus is a prevalent soil fungus that is vital in soil ecology and crop health. It facilitates the absorption of mineral elements by plant roots while inhibits pathogens (Miao et al., 2016; Li et al., 2018). A subset of saprophytic bacteria within the Ascomycota phylum is crucial in decomposing recalcitrant organic matter present in soil, thereby facilitating nutrient cycling. However, certain members of Ascomycota can also induce plant diseases such as root rot, stem rot, fruit rot and branch blight (Beimforde et al., 2014; Zhao et al., 2019).

The concentration of ozone in nutrient solutions may also impact crop growth and yield. Studies have shown that high levels of ozone can damage crops and vegetation (Rai et al., 2015) and can adversely affect plant growth, active ingredients and yield (Lee et al., 2022). For example, high concentrations of ozone can reduce the biomass of potatoes, cause leaf damage, and decrease the total number of potato tubers. Increased ozone concentrations can also impact the diversity of root-associated bacteria in crops (Feng et al., 2015). For instance, O_3_ has inhibitory effects on the microbial community in wheat rhizosphere soil (Li et al., 2012) and indirectly affects the composition of microbial communities in poplar rhizosphere soil negatively (Wang et al., 2024). An increase in O_3_ concentration can result in a decline in the biomass of fungi and gram-negative bacteria (Díaz-López et al., 2022), thereby perturbing the microbial community composition, particularly by reducing nitrifying bacteria and enhancing the relative abundance of fungal groups capable of denitrification. Elevated O_3_ not only impacts the structure of soil microbial communities but also has potential implications for their functional capabilities (Chen et al., 2019). In this study, a saturated oxygen and ozone micro/nanobubble nutrient solution was used. However, there is no clear categorization of the dissolved oxygen mass concentration in micro/nanobubble nutrient solutions, and further investigation is needed to determine the optimal gas concentration promoting plant growth and optimizing microbial community of substrate. We aimed to develop an irrigation frequency that is more suitable for meeting the requirements of plant growth while also being cost-effective, scientifically sound, and sustainable.

## 5 Conclusion

In summary, the application of micro/nanoscale (O_2_ and O_3_) bubble nutrient solution can promote photosynthesis and increase the yield of substrate-cultured lettuce plants. The majority physical and chemical indexes of the substrate also exhibited an increasing trend, while SC and UE activities decreased. The change in the rhizosphere microbial community with O_2_ and O_3_ treatment revealed that both the abundance and diversity of bacterial and fungal communities in the substrate increased after plant cultivation and decreased following treatment with micro/nanobubble nutrient solutions. The O_3_ group had a more pronounced impact on the microbial community structure in the substrate than the O_2_ group. Our study provides a method that combines micro/nanobubble generation technology with a soilless substrate to cultivate lettuce plants, which can promote robust seedling growth and substantially increase lettuce yield.

## Data availability statement

The raw sequencing data have been deposited in the NCBI Sequence Read Archive (SRA) under accession number PRJNA1078579.

## Author contributions

QZ: Data curation, Funding acquisition, Investigation, Methodology, Validation, Writing – original draft. JD: Formal analysis, Funding acquisition, Validation, Writing – original draft, Writing – review & editing. SL: Data curation, Investigation, Writing – original draft. WL: Formal analysis, Writing – original draft. AL: Conceptualization, Visualization, Writing – review & editing.

## Supplementary material

Supplementary Figure 1 Rarefaction curves and ASV rank-abundance curves of different substrate samples. **(A)** Rarefaction curves of bacterial communities. **(B)** Rarefaction curves of fungal communities. **(C)** ASV rank-abundance curves of the bacterial communities. **(D)** ASV rank-abundance curves of fungal communities. The horizontal coordinate is the sequencing depth, and the vertical coordinate is the median value of the α diversity index calculated 10 times, which is presented in a box plot.

Supplementary Table 1 The mother liquor formula of Japanese Yamazaki nutrient solution for lettuce.

Supplementary Table 2 Sequencing information for different samples.
