# Supplementary figures and images for: Effects of micro/nano-ozone bubble nutrient solutions on growth promotion and rhizosphere microbial community diversity in soilless cultivated lettuces
Source: Front Plant Sci. 2024 Apr 11;15:1393905. doi: 10.3389/fpls.2024.1393905 (PMC11043558; doi:10.3389/fpls.2024.1393905)

**A**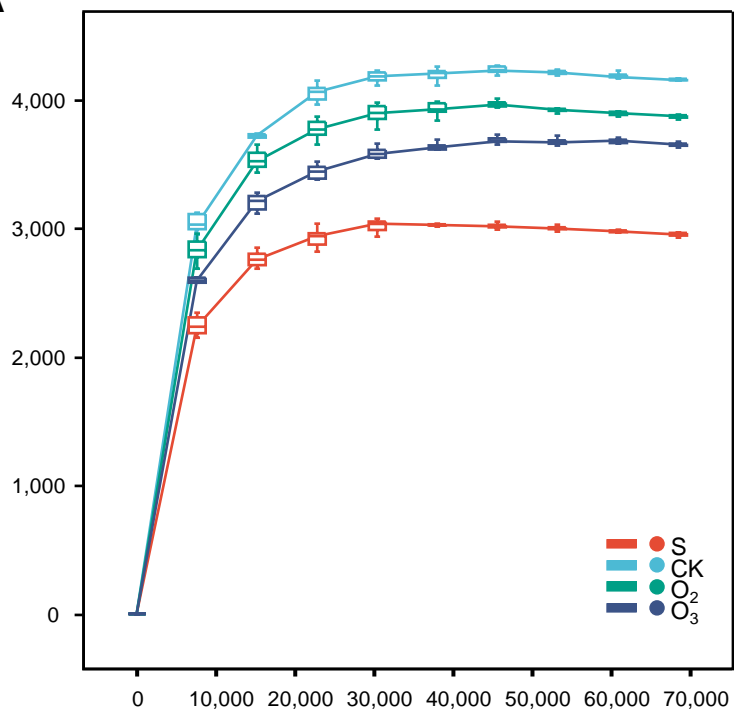**B**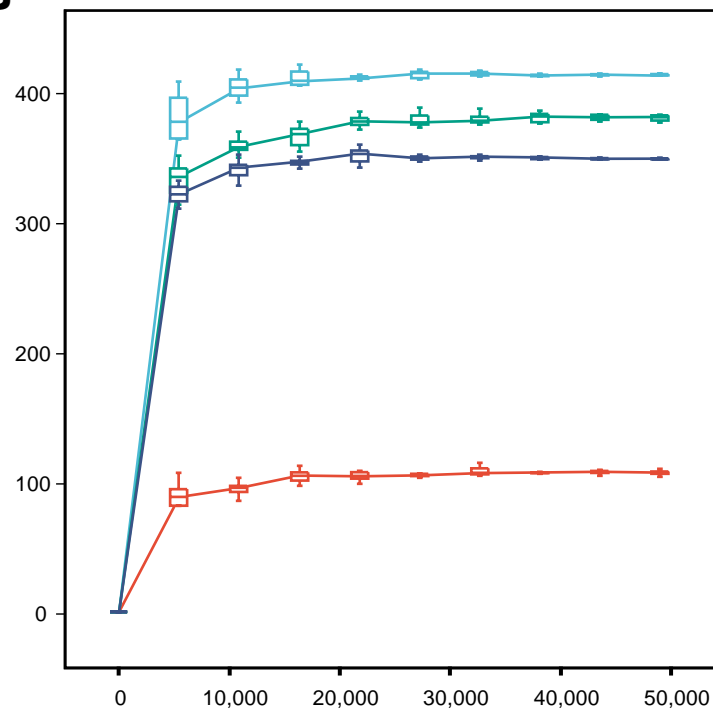**C**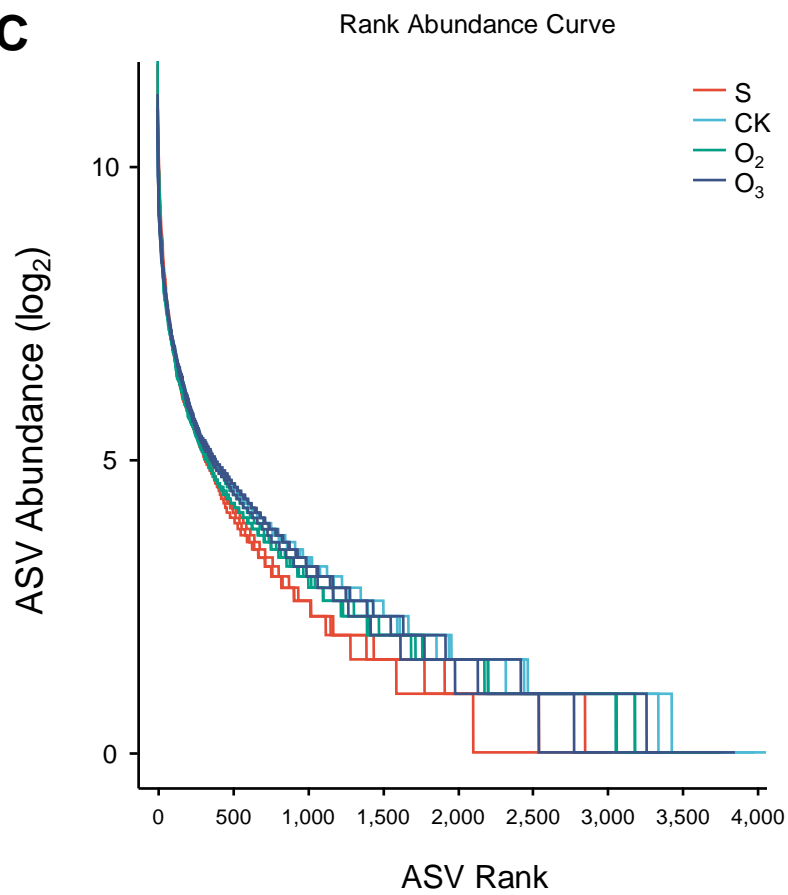**D**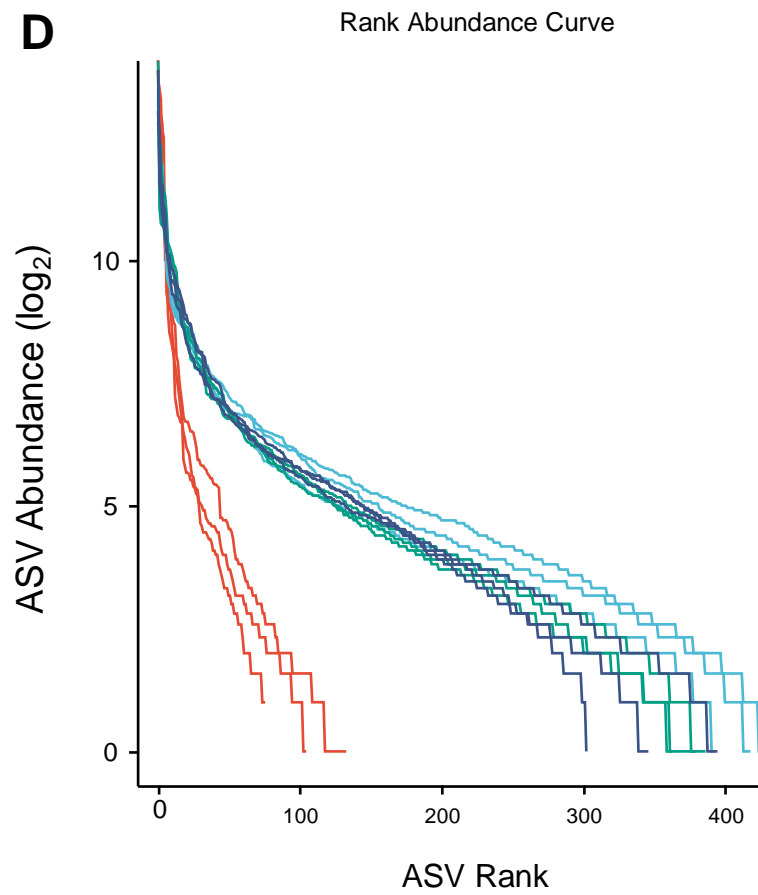

Supplement: Supplementary Figure 1 — Rarefaction curves and ASV rank-abundance curves of different substrate samples. (A) Rarefaction curves of bacterial communities. (B) Rarefaction curves of fungal communities. (C) ASV rank-abundance curves of the bacterial communities. (D) ASV rank-abundance curves of fungal communities. The horizontal coordinate is the sequencing depth, and the vertical coordinate is the median value of the α diversity index calculated 10 times, which is presented in a box plot. [file DataSheet_1.pdf]
