# Supplementary material for: Effects of micro/nano-ozone bubble nutrient solutions on growth promotion and rhizosphere microbial community diversity in soilless cultivated lettuces
Source: Front Plant Sci. 2024 Apr 11;15:1393905. doi: 10.3389/fpls.2024.1393905 (PMC11043558; doi:10.3389/fpls.2024.1393905)
Supplement: Supplementary Table 1 — The mother liquor formula of Japanese Yamazaki nutrient solution for lettuce. [file Table_1.docx]

**Table S1.** The mother liquor formula of Japanese Yamazaki nutrient solution for lettuce

| Nutrient solution category | Salt compound | Dosage (mg/L) |
| --- | --- | --- |
| A solution | Calcium nitrate tetrahydrate | 23600 |
|  | Potassium nitrate | 40400 |
| B solution | Ammonium dihydrogen phosphate | 5700 |
|  | Magnesium sulfate heptahydrate | 12300 |
| C solution | Boric acid | 2860 |
|  | Manganese sulfate heptahydrate | 2130 |
|  | Zinc sulfate heptahydrate | 220 |
|  | Copper sulfate anhydrous | 80 |
|  | Ammonium molybdate | 20 |
|  | Ethylenediaminetetraacetic acid iron (Ⅲ) sodium salt | 20000 |

**Note**: **Solution A**: 23.6g calcium nitrate tetrahydrate were added into a beaker with 500 mL water; After it is fully dissolved, 40.4g potassium nitrate were added and set the volume to 1 L after it is fully dissolved.

**Solution B**: 5.7 g ammonium dihydrogen phosphate were added into a beaker with 500 mL water and dissolved adequately; then 12.3 g magnesium sulfate heptahydrate were added; After dissolution, the volume is fixed to 1L.

**Solution C**: 0.286 g boric acid were dissolved into 50 mL water. After it is fully dissolved, 0.213 g manganese sulfate heptahydrate, 0.022 g zinc sulfate heptahydrate, 0.008g copper sulfate anhydrous, and 0.002 g ammonium molybdate were added. Finally, 2 g of ethylenediaminetetraacetic acid iron (Ⅲ) sodium salt were added, After dissolution, the volume is fixed to 100 mL.

It needs to be dissolved by heating during preparation. When used, the mother liquor is mixed according to the ratio of nutrient solution A: B: C=1:1:0.1, and then diluted to 100 times for use.
